# Interactions of Polymyxin B in Combination with Aztreonam, Minocycline, Meropenem, and Rifampin against Escherichia coli Producing NDM and OXA-48-Group Carbapenemases

**DOI:** 10.1128/AAC.01065-21

**Published:** 2021-11-17

**Authors:** Anna Olsson, Marcus Hong, Hissa Al-Farsi, Christian G. Giske, Pernilla Lagerbäck, Thomas Tängdén

**Affiliations:** a Department of Medical Sciences, Uppsala University, Uppsala, Sweden; b Department of Laboratory Medicine, Division of Clinical Microbiology, Karolinska Institute, Stockholm, Sweden; c Department of Clinical Microbiology, Karolinska University Hospital, Stockholm, Sweden

**Keywords:** carbapenem resistance, Gram-negative bacteria, combination therapy, synergy, polymyxins

## Abstract

Carbapenemase-producing *Enterobacterales* pose an increasing medical threat. Combination therapy is often used for severe infections; however, there is little evidence supporting the optimal selection of drugs. This study aimed to determine the *in vitro* effects of polymyxin B combinations against carbapenemase-producing Escherichia coli. The interactions of polymyxin B in combination with aztreonam, meropenem, minocycline or rifampin against 20 clinical isolates of NDM and OXA-48-group-producing E. coli were evaluated using time-lapse microscopy; 24-h samples were spotted on plates with and without 4× MIC polymyxin B for viable counts. Whole-genome sequencing was applied to identify resistance genes and mutations. Finally, potential associations between combination effects and bacterial genotypes were assessed using Fisher's exact test. Synergistic and bactericidal effects were observed with polymyxin B and minocycline against 11/20 strains and with polymyxin B and rifampin against 9/20 strains. The combinations of polymyxin B and aztreonam or meropenem showed synergy against 2/20 strains. Negligible resistance development against polymyxin B was detected. Synergy with polymyxin B and minocycline was associated with genes involved in efflux (presence of *tet[B]*, wild-type *soxR*, and the *marB* mutation H44Q) and lipopolysaccharide synthesis (*eptA* C27Y, *lpxB* mutations, and *lpxK* L323S). Synergy with polymyxin B and rifampin was associated with sequence variations in *arnT*, which plays a role in lipid A modification. Polymyxin B in combination with minocycline or rifampin frequently showed positive interactions against NDM- and OXA-48-group-producing E. coli. Synergy was associated with genes encoding efflux and components of the bacterial outer membrane.

## INTRODUCTION

The increasing prevalence of carbapenemase-producing *Enterobacterales* is an emerging threat worldwide. These bacteria are common causes of severe infections, such as sepsis, urinary tract infections, and hospital-acquired pneumonia, and are difficult to treat due to their multidrug-resistant phenotypes (1–3). The last resort antibiotics polymyxin B and E (colistin) remain active against most isolates and have been widely used for these infections (4, 5). Although combination therapy is always recommended based on observational clinical data (6), evidence is still scarce on the optimal selection of companion drug.

*In vitro* synergy against carbapenemase-producing *Enterobacterales* has been shown with polymyxins in combination with multiple other antibiotics (e.g., β-lactams, minocycline, rifampin) (7–10). Most studies have addressed Klebsiella pneumoniae, and data are limited for Escherichia coli. The prevailing theory for the observed synergistic interactions is that the polymyxin-induced membrane disruption increases the membrane permeability, thereby facilitating entry of the second antibiotic (11, 12). Polymyxins may also act by counteracting the function of membrane-associated efflux pumps (11). However, the mechanisms of synergistic interaction remain largely unknown. Therefore, to date, the activity of antibiotic combinations cannot be predicted based on antibiotic susceptibility testing of single drugs or genetic characterization.

We previously evaluated automated time-lapse microscopy (the oCelloScope, BioSense Solutions Aps, Farum, Denmark) as a screening tool for antibiotic combinations (13) and reported synergy with several polymyxin B combinations against multidrug-resistant K. pneumoniae and Pseudomonas aeruginosa (9, 14). In the present study, we evaluated the effects of polymyxin B in combination with aztreonam, meropenem, minocycline and rifampin against 20 NDM- and OXA-48-producing E. coli in 24-h time-lapse microscopy experiments. A spot assay in which 24-h samples were placed on plates with and without polymyxin B at 4× MIC was added to provide viability data and detect emerging subpopulations with reduced susceptibility. All isolates were subjected to whole-genome sequencing to map genes known to impact the susceptibility to the tested antibiotics. Finally, we explored potential associations between the observed combination effects and bacterial genetics.

## RESULTS

### Antibiotic susceptibilities.

All strains were intermediate to polymyxin B with MICs of 0.5 mg/liter (Table 1). Only three strains were susceptible to aztreonam. Strains carrying *bla*_NDM_ (*bla*_NDM-1_, *bla*_NDM-5_ and *bla*_NDM-7_) were resistant to meropenem, whereas those carrying only *bla*_OXA-48_ –group carbapenemase genes (*bla*_OXA-48_ and *bla*_OXA-181_) were classified as susceptible. Minocycline MICs varied greatly between the strains (range 1–64 mg/liter) and rifampin MICs were mostly high (8 to 32 mg/liter).

**TABLE 1:** MIC values (mg/liter) and classification of antibiotic susceptibilities according to CLSI breakpoint tables M100-ED30:2020^*a*^

Strain	Carbapenemase	Polymyxins	β-lactams		Tetracyclines	Rifamycins
		PMB	ATM	MEM	MIN	RIF
ARU770	NDM-1	0.5 (I)	>16 (R)	>64 (R)	32 (R)	16 (NA)
ARU771	NDM-1	0.5 (I)	>16 (R)	64 (R)	32 (R)	16 (NA)
ARU772	NDM-7	0.5 (I)	>16 (R)	32 (R)	4 (S)	16 (NA)
ARU773	NDM-5	0.5 (I)	1 (S)	64 (R)	16 (R)	16 (NA)
ARU774	NDM-1	0.5 (I)	>16 (R)	>64 (R)	16 (R)	32 (NA)
ARU775	NDM-5	0.5 (I)	>16 (R)	>64 (R)	4 (S)	16 (NA)
ARU776	NDM-1	0.5 (I)	>16 (R)	>64 (R)	4 (S)	16 (NA)
ARU777	NDM-5	0.5 (I)	>16 (R)	16 (R)	16 (R)	16 (NA)
ARU778	NDM-1	0.5 (I)	>16 (R)	16 (R)	16 (R)	32 (NA)
ARU779	NDM-5	0.5 (I)	>16 (R)	>64 (R)	8 (I)	16 (NA)
ARU780	NDM-5	0.5 (I)	8 (I)	64 (R)	8 (I)	16 (NA)
ARU781	NDM-5	0.5 (I)	>16 (R)	>64 (R)	8 (I)	16 (NA)
ARU782	NDM-5	0.5 (I)	>16 (R)	64 (R)	4 (S)	32 (NA)
ARU783	OXA-48	0.5 (I)	≤0.5 (S)	0.5 (S)	2 (S)	8 (NA)
ARU785	OXA-48	0.5 (I)	>16 (R)	2 (S)	1 (S)	8 (NA)
ARU786	OXA-48	0.5 (I)	≤0.5 (S)	1 (S)	8 (I)	16 (NA)
ARU787	OXA-181	0.5 (I)	>16 (R)	1 (S)	8 (I)	32 (NA)
ARU788	OXA-181	0.5 (I)	>16 (R)	0.5 (S)	16 (R)	16 (NA)
ARU790	NDM-5, OXA-181	0.5 (I)	>16 (R)	16 (R)	32 (R)	32 (NA)
ARU791	NDM-1, OXA-48	0.5 (I)	>16 (R)	32 (R)	64 (R)	32 (NA)

a Abbreviations: S, susceptible; I, intermediate; R, resistant; NA, not available; ATM, aztreonam; MEM, meropenem; MIN, minocycline; PMB, polymyxin B; RIF, rifampin

### Resistance genes and mutations.

Polymyxin resistance genes *mcr-1 – 10* were not found in the strains. All strains harbored genes encoding carbapenemases: NDM (*n* = 13), OXA-48-group enzymes (*n* = 5) or both (*n* =2) (Table 2). In addition, other β-lactamase genes were present in all strains, most frequently *bla*_TEM-1B_ (*n* = 15), *bla*_CTX-M-15_ (*n* = 14) and *bla*_OXA-1_ (*n* = 11). Tetracycline efflux genes *tet(A*) (*n* = 8), *tet(B)* (*n* = 8) or *tet(D)* (*n* = 2) were found in 18/20 strains. All eight strains harboring *tet(B)* and eight of nine strains with wild type *soxR* (Table 3) had increased minocycline MICs (≥8 mg/liter). An amino acid substitution in *rpoB* (G1261C) was identified in ARU790 but was not located in any region known to cause resistance to rifampin (15).

**TABLE 2:** Identified resistance genes and amino acid variations^*a*^

		Strain																			
Antibiotic class	Resistance gene	ARU770	ARU771	ARU772	ARU773	ARU774	ARU775	ARU776	ARU777	ARU778	ARU779	ARU780	ARU781	ARU782	ARU783	ARU785	ARU786	ARU787	ARU788	ARU790	ARU791
β-lactams	*bla*_CMY-2_											+							+	+	
	*bla*_CMY-6_									+											
	*bla*_CMY-42_							+								+		+			
	*bla*_CTX-M-15_	+	+	+		+	+	+	+				+	+		+		+	+	+	+
	*bla*_NDM-1_	+	+			+		+		+											+
	*bla*_NDM-5_				+		+		+		+	+	+	+						+	
	*bla*_NDM-7_			+																	
	*bla*_OXA-1_			+		+	+	+	+	+			+	+				+	+	+	
	*bla*_OXA-9_	+																			
	*bla*_OXA-48_														+	+	+				+
	*bla*_OXA-181_																	+	+	+	
	*bla*_TEM-1B_	†	+		+	+	+	+		+	†	+	+	+	+		+	+	+	+	+
Tetracyclines	*tet(A)*			+		+	+	+					+	+	+			+			
	*tet(B)*	+	+		+				+	+	+								+	+	
	*tet(D)*																+				+
Rifamycins	*rpoB* ^*b*^	+	+	+	+	+	+	+	+	+	+	+	+	+	+	+	+	+	+	G1261C	+

a Abbreviations: +, full-length gene present and without amino acid variations compared to the reference in the ResFinder database; †, gene sequence not complete due to scaffold or contig-breaks after assembly.

b Escherichia coli K12 MG1655 used as reference (NCBI accession number: NC_000913.3).

**TABLE 3:** Genetic differences^*a*^ in genes encoding porins, efflux pumps, and their regulators

Porin, efflux pump or regulator	Function	Gene	Strain									
			**ARU770**	**ARU771**	**ARU772**	**ARU773**	**ARU774**	**ARU775**	**ARU776**	**ARU777**	**ARU778**	**ARU779**
OmpC	S	*ompC* ^*b*^	Q54K	Q54K	L296V	Q54K	Q54K	Q54K	Q54K		G216A	Q54K
			N165D	N165D		N165D	N165D	N165D	N165D		I218V	N165D
			G216A	G216A		G216A	G216A	G216A	G216A			G216A
			I218V	I218V		I218V	I218V	I218V	I218V			I218V
OmpF	S	*ompF* ^*b*^	N31fs	N31fs								Y112F
												F118I
												Y204F
												A233fs
AcrAB-To1C	S	*acrA*									T104A^*d*^	T104A^*d*^
											A167S^*d*^	
	S	*acrB*									H596N^*d*^	H596N^*d*^
	R	*acrR*	T5N^*c*^	T5N^*c*^			V29fs	V29fs	V29fs			K80fs
	S	*tolC*								Y120H		
*marRAB* operon	R	*marR*	G103S^*d*^	G103S^*d*^	G103S^*d*^	G103S^*d*^					S3N^*d*^	†
			Y137H^*d*^	Y137H^*d*^	Y137H^*d*^	Y137H^*d*^					G103S^*d*^	
											Y137H^*d*^	
	A	*marA*					†			†	S127N	
	R	*marB*	H44Q	H44Q	H44Q	H44Q				X	S5L	S5L
											A10T	L12F
											A33G	A17T
											H44Q	V20I
												H44Q
SoxSR	A	*soxS*										
	A	*soxR*					A111T^*d*^	A111T^*d*^	A111T^*d*^	A111T^*d*^	G74R^*d*^	T38S^*d*^
												G74R^*d*^
RobA	A	*rob*									Q20H	
											A171S	
OmpR-EnvZ	A	*ompR*										
	A	*EnvZ*									A25V	A25V
											T466A	T466A
			**ARU780**	**ARU781**	**ARU782**	**ARU783**	**ARU785**	**ARU786**	**ARU787**	**ARU788**	**ARU790**	**ARU791**
OmpC	S	*ompC* ^*b*^	G216A	L296V	Q54K	M57V	Q54K	Q54K	Q54K	L296V	L296V	G216A
			I218V		N165D	G216A	N165D	N165D	N165D			I218V
					G216A	I218V	G216A	G216A	G216A			L296V
					I218V	L296V	I218V	I218V	I218V			
OmpF	S	*ompF* ^*b*^					Y112F	Y112F				S199fs
							F118I	F118I				
							Y204F	Y204F				
AcrAB-TolC	S	*acrA*				T104A^*d*^	T104A^*d*^	T104A^*d*^				
						N221Y^*d*^						
	S	*acrB*				H596N^*d*^	H596N^*d*^	H596N^*d*^				K1036T
	R	*acrR*	S87*	S68*	V29fs				V29fs			V43fs
	S	*tolC*				A440T						
*marRAB* operon	R	*marR*	A70E	G103S^*d*^		G103S^*d*^	K62R^*d*^	K62R^*d*^		G103S^*d*^	†	X
			G103S^*d*^	Y137H^*d*^		Y137H^*d*^	Δ97-107	G103S^*d*^		Y137H^*d*^		
			Y137H^*d*^				Y137H^*d*^	Y137H^*d*^				
	A	*marA*										
	R	*marB*	H44Q	S5L		S5L	S5L	S5L		H44Q	H44Q	H44Q
				T24P		A17T	L12F	L12F				
				A33G		V20I	A17T	A17T				
				V38A		H44Q	V20I	V20I				
				H44Q			H44Q	H44Q				
SoxSR	A	*soxS*										A12S^*c*^
	A	*soxR*			A111T^*d*^	T38S^*d*^	T38S^*d*^	T38S^*d*^	A111T^*d*^			
						G74R^*d*^	G74R^*d*^	G74R^*d*^				
RobA	A	*rob*										
OmpR-EnvZ	A	*ompR*										
	A	*EnvZ*				A25V	A25V	A25V				
						T466A	T466A	T466A				

a Escherichia coli K12 MG1655 (NCBI accession number: NC_000913.3) used as reference. Abbreviations: A, activator; R, repressor; S, subunit; X, gene not found; *, stop codon; fs, frameshift; †, gene sequence not complete due to scaffold or contig-breaks after assembly.

b Only amino acid variations in β-strand-encoding regions of *ompC* and *ompF* are shown.

c Mutations previously known to cause increased resistance.

d Mutation not previously associated with increased efflux.

Eleven strains had a sequence variation (T5N, *n* = 2), frameshift (*n* = 7) or a premature stop codon (*n* = 2) in *acrR* (Table 3). These genetic variations likely result in increased expression of the AcrAB-TolC efflux pump (16), for which aztreonam, meropenem, minocycline and rifampin are known substrates (17–19). A mutation in the AcrAB-TolC efflux regulatory gene *soxS* (A12S), previously reported to be associated with resistance, was found in one strain (20). We identified additional mutations commonly encountered in clinical isolates but have not been shown to increase AcrAB-TolC efflux activity alone: *acrA* (T104A, A167S and N221Y [21]), *marR*, (S3N, K62R, G103S and Y137H) and *soxR* (A111T, T38S and G74R) (22). Several other mutations with unknown effects were found in *marB*; the most frequent mutation was H44Q which was found in 14/20 strains. In 19/20 strains, genes encoding the OmpC and OmpF porins, that facilitate entry of β-lactams (3), were associated with sequence variations in the β-sheet regions composing the porin channels (23, 24) (Table 3). Several amino acid variations were identified in genes encoding enzymes involved in the synthesis or modification of LPS, mainly in *lpxB*, *lpxK*, *lpxH, arnT*, and *eptA* (25) (Table 4). Moreover, there was large variability in core oligosaccharide types, as determined based on the *waa* locus (25, 26); R1 was most frequent (*n* = 8), followed by R4 (*n* = 5), R2 (*n* = 4) and R3 (*n* = 3).

**TABLE 4:** Genetic differences^*a*^ in genes encoding enzymes involved in lipopolysaccharide synthesis and core oligosaccharide type

	Gene	Strain																				
Function		ARU770	ARU771	ARU772	ARU773	ARU774	ARU775	ARU776	ARU777	ARU778	ARU779	ARU780	ARU781	ARU782	ARU783	ARU785	ARU786	ARU787	ARU788	ARU790	ARU791	
Structural component	*lpp*										R77H											
Enzymes catalyzing lipid A synthesis	*lpxA*																					
	*lpxC*																					
	*lpxD*									R206C	I224V				K147R	K147R	K147R					
										I224V					I224V	I224V	I224V					
	*lpxH*	K58Q	K58Q	K58Q	K58Q					V13A	V13A	K58Q			V13A	V13A	V13A		K58Q	K58Q	K58Q	
				T139M	T139M					R57H	K58Q				K58Q	K58Q	K58Q		T139M	T139M		
				W190G						K58Q						R109Q			W190G	W190G		
										P210T												
	*lpxB*	E3K	E3K	E3K	H32R					H32R	H32R	R220C	H32R		H32R	H32R	H32R		E3K	E3K	H32R	
				H32R	V93I					A180G	A180G		A180G		K84T	A180G	A180G		H32R	H32R		
										H189Y	L256M		H189Y		A180G	H189Y	H189Y					
										E247A	M260L				L256M	L256M	L256M					
	*lpxK*	A278T	A278T	L323S	Y93N					D118G	T319A	L323S	Q74K		D118G	V66I	V66I		L323S	L323S	L323S	
		L323S	L323S		A278T					T319A	L323S		L323S		D225E	D118G	D118G					
					L323S					L323S					T319A	R293L	R293L					
										N328H					L323S	A294P	A294P					
																E298G	E298G					
																T319A	T319A					
																L323S	L323S					
	*lpxL*									M254I											M185T
	*lpxM*	†								A98V												
Core oligosacharide type	-	R3	R3	R1	R4	R1	R2	R1	R2	R1	R4	R1	R1	R2	R4	R4	R4	R2	R1	R1	R3	
Regulators of lpxC	*ftsH*																				
	*lapB*														T201A	T201A	T201A				†
															D323N	T366A	T366A				
															E331K						
Modifies lipid A	*arnT*	T116A	T116A		T116A				T116A	T33M	T116A		T116A		T116A	T116A	T116A				N232D
		T197S	T197S		T197S				T197S	D108N	T197S		T197M		N232D	T197S	T197S				
		N232D	N232D		N232D				N232D	N232D	N232D		N232D		V261L	N232D	N232D				
		L248M	L248M		L248M				L248M	V261L	L248M		V261L		T281A	L248M	L248M				
		V261L	V261L		V261L				V261L	T281A	V261L		T281A		S322P	V261L	V261L				
		T281A	T281A		T281A				T281A	S322P	T281A				P408S	T281A	T281A				
		S322P	S322P		S322P				S322P	A535V	S322P					S322P	S322P				
		P408S	P408S		P408S				P408S		P408S					P408S	P408S				
		D522N	D522N		D522N				D522N		D522N					D522N	D522N				
	*eptA*	D348G	D348G	D348G	D348G	C27Y	C27Y	C27Y	C27Y	L14F	A147T	E547K	E547K	C27Y	D348G	K233T	K233T	C27Y	D348G	D348G	V320Del
		T413S	T413S	T413S	T413S					A15S	D348G					D348G	D348G		T413S	T413S	VLWND
										A21T											NDGGC
										I26V											KGACD
										A39V											RVPHQ
										S69G											NVTAL
										A106T											NLPDQ
										Q123R											CIN
										F130L											T413S
										L137I											
										A147T											
										V163I											
										L211S											
										V217I											
										E232G											
										A332V											
										D348G											
										E366D											
										K414Q											
	*pagP*									S11F	N2I				L82Q	K5Q	K5Q				L82Q
										L82Q	R35G					L82Q	L82Q				
										T117I	A39T										
	*lpxP*				†		G125F		Q4K	A46T	Q4K				M47I	Q4K	G192S				
									F253Y	F253Y	T263A				F253Y	F253Y	F253Y				
									T263A	E73K G187S F253Y					T263A	T263A	T263A				

a Escherichia coli K-12 MG1655 (NCBI accession number: NC_000913.3) used as reference. Abbreviations: Del, deletion; †, gene sequence not complete due to scaffold or contig-breaks after assembly.

### Time-lapse microscopy experiments.

**FIG 1:**
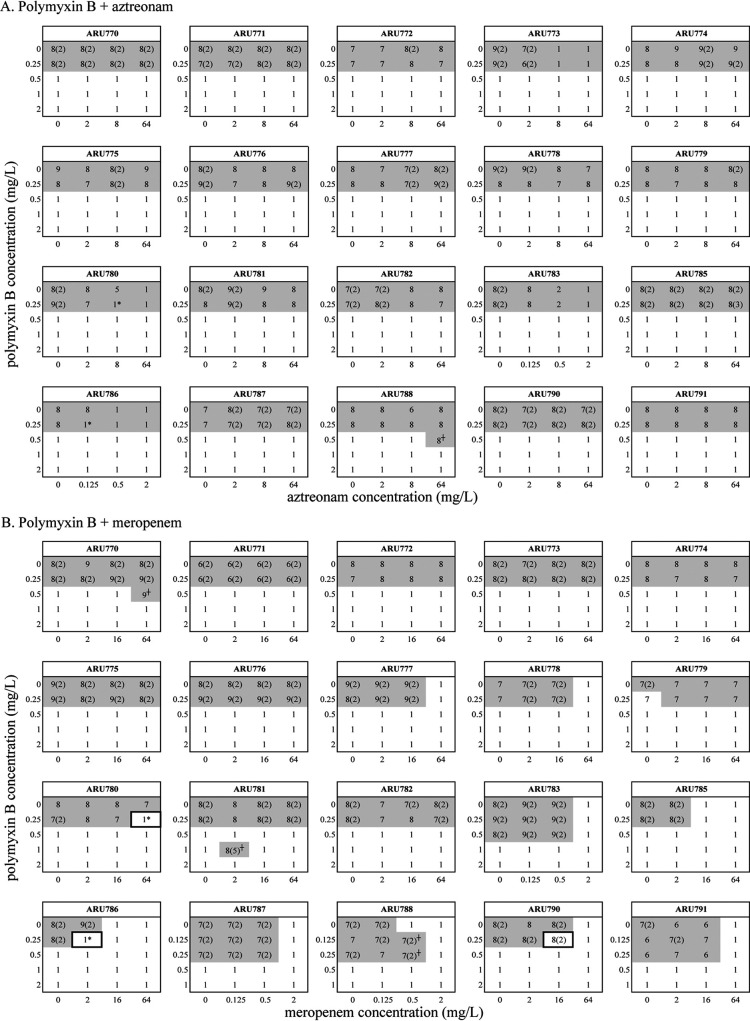
Results of time-lapse microscopy experiments and spot assay. For time-lapse microscopy experiments, wells with BCA >8 and SESA_max_ >5.8, indicating a bacterial density of >10^6^ CFU/ml at 24 h, are highlighted in gray and combinations showing positive interactions in the time-lapse microscopy experiments are marked with a square. For spot assay, bacterial growth on MH-II plates at 24 h is presented in log_10_ CFU/ml and no visible growth is set to 1 log_10_ CFU/ml (LOD = 2 log_10_ CFU/ml). Growth on 4× MIC polymyxin B is presented in parentheses. Synergistic and bactericidal effect with the combination, as determined with the spot assay, is highlighted with “*” and antagonistic effect “^+^.”

The most effective combination was polymyxin B and minocycline, showing a positive interaction against 11/20 strains (Fig. 1), closely followed by polymyxin B and rifampin with 9/20 strains. For polymyxin B and meropenem a positive interaction was seen against 3/20 strains. The combination of polymyxin B and aztreonam was not superior to monotherapy at any of the tested concentrations when using the predefined cutoffs for bacterial growth (BCA >8 at 24 h and SESA_max_ >5.8). Negative interaction by the combination in comparison to monotherapy was observed with polymyxin B in combinations with meropenem (ARU770, ARU779, ARU781 and ARU788) and aztreonam (ARU788).

### Spot assay.

The spot assay showed synergistic and bactericidal effects with 22/23 combinations that indicated positive interactions in the time-lapse microscopy experiments (Fig. 1). In addition, synergistic and bactericidal effects were detected with polymyxin B and aztreonam against two strains (ARU780 and ARU786). No antibiotic carryover effect was observed (data not shown). Growth on polymyxin B at 4× MIC after 24 h was detected for 267 of the 504 spots (53%) that grew on nonantibiotic-containing plates (Fig. 1). However, in all but three cases, growth on 4× MIC polymyxin B was only 2 log_10_ CFU/ml (= the lower limit of detection, LOD) and repeated susceptibility testing of 67 spots revealed no increase in polymyxin B MICs indicating an inoculum effect (data not shown).

### Associations between combination effects and bacterial genetics.

Statistical analysis using Fisher’s exact text showed that synergy with polymyxin B and minocycline was significantly associated with the tetracycline efflux gene *tet(B)*; synergy was noted in 7/8 strains carrying this gene (*P* = 0.0281) (Table S1). In contrast, a negative association was found for *tet(A)*; synergy was only observed in 1/8 harboring this gene (*P* = 0.0045). Statistically significant associations were also found when comparing wild type to any mutation(s) in *marB* (*P* = 0.0081), *marR* (*P* = 0.0499) and *soxR (P* = 0.0098), which are all involved in AcrAB-TolC efflux. On the mutation level, the *marB* mutation H44Q was frequently associated with a synergistic effect (10/11, *P* = 0.04985) (Table S2). No specific *marR* mutation was significantly associated with synergy. Reduced susceptibility to minocycline in strains carrying *tet(B) (n *= 8) or wild type *soxR (n *= 9) was reversed in the presence of polymyxin B in 7 and 8 cases, respectively (Fig. 1C). In contrast, the *soxR* mutation A111T was negatively associated with synergy (1/11, *P* = 0.0499). Moreover, sequence alterations in the *lpxB* (*P* = 0.0499) and *lpxK* (*P* = 0.0499) genes, encoding enzymes involved in lipid A synthesis, were associated with synergy (Table S3) (25). On the mutation level, the *lpxK* mutation L323S (*P* = 0.0499) was present in 10/11 strains against which synergy was found, whereas the *eptA* mutation C27Y showed a negative association (1/11, *P* = 0.0499).

No significant associations were noted for the polymyxin B and rifampin combination for genes encoding efflux, porin loss or enzymatic resistance. However, several mutations in the *arnT* gene encoding a lipid A-modifying enzyme were positively associated with synergy (*P values* ranging from 0.005 to 0.022) (Table S4). Because synergy was rarely observed with polymyxin B and aztreonam or meropenem, statistical analyses were not considered meaningful for these combinations.

## DISCUSSION

In this study, positive interactions were frequently found with polymyxin B combined with minocycline or rifampin against NDM- and OXA-48-group producing E. coli. In contrast, according to the 24-h viable count data, combinations of polymyxin B and aztreonam or meropenem showed synergy and a bactericidal activity only against 2/20 strains. Negligible resistance development against polymyxin B was identified with all combinations. Although growth on polymyxin B at 4× MIC was often observed following antibiotic exposure, bacterial concentrations were typically low (≤2 log_10_ CFU/ml) and no MIC elevations were detected. Therefore, we deduce that this observation likely reflects an inoculum effect, which is of uncertain clinical relevance, rather than emergence or selection of resistant subpopulations.

Importantly, nonsusceptibility to one or both constituent antibiotics does not preclude a synergistic activity when combining the two drugs. Polymyxin B and minocycline performed well in this study despite that all strains were intermediate to polymyxin B, and most were intermediate or resistant to minocycline. To our knowledge, data on the activity of this combination against *Enterobacterales* are scarce. However, polymyxin B was previously reported to induce 8-fold reductions in minocycline MICs in *mcr-1* positive E. coli and K. pneumoniae (8). Also, we recently reported synergy with this combination in time-kill experiments against 4/5 K. pneumoniae producing NDM, KPC or OXA-48 enzymes, including strains displaying phenotypic resistance to one or both drugs (9).

Gram-negative bacteria are intrinsically resistant to rifampin due to the inability of this molecule to penetrate the bacterial outer membrane. Yet, polymyxin B and rifampin showed synergy against 9/20 strains in this study. Our results are consistent with other studies reporting positive interactions with polymyxins and rifampin. One study observed a bactericidal activity with polymyxin B and rifampin against 2/5 KPC-producing E. coli (10) and we previously reported synergy with this combination against 4/5 NDM-, KPC- or OXA-48-producing K. pneumoniae (9). Another study showed synergy with this combination against NDM- and MCR-1-producing polymyxin-resistant E. coli (7).

Our results indicate polymyxin B and meropenem has low synergistic potential against NDM- and OXA-48-producing E. coli. Polymyxin-carbapenem combinations have been widely recommended for severe infections caused by carbapenemase-producing *Enterobacterales* (4, 6). Observational clinical data support the use of such combinations against KPC-producing K. pneumoniae with carbapenem MICs ≤8 mg/liter (4, 5). However, their efficacy against E. coli and strains producing non-KPC enzymes remains uncertain as illustrated in this study. As new β-lactam/β-lactamase inhibitor combinations become available, it is important to consider the bacterial genetic determinants and strain-dependent differences in antibiotic susceptibility to the single drugs and combinations. While meropenem-vaborbactam and imipenem-relebactam are normally active against KPC-producing isolates (6), their use will be limited in areas where other carbapenemases are predominant. Aztreonam is highly intriguing in this context due to its stability to metallo-β-lactamases, such as NDM-1. Still, polymyxin B and aztreonam failed to show positive interactions against most of the tested strains in this study. To our knowledge, previous data on this combination is lacking for E. coli and is scarce for K. pneumoniae (9, 27). Clearly, coadministration of polymyxin B was generally not sufficient to circumvent enzymatic resistance in these strains, e.g., mediated by CTX-M-15, which was produced by 14/20 strains and has high affinity for aztreonam (2, 28).

We observed several biologically plausible and statistically significant associations between the interactions of polymyxin B and minocycline and bacterial genetics. For example, synergy was positively associated with genes involved in efflux, which can be counteracted by the membrane-disrupting activity of polymyxin B. Statistically significant associations were observed for mutant *marB* and *marR*. These genes regulate AcrAB-TolC efflux, for which minocycline and multiple antibiotics (e.g., meropenem, aztreonam and rifampin) are known substrates. The association with the *marB* mutation H44Q likely results from reduced repression of *marA*, which in turn increases AcrAB-TolC activity (1) (Table S2). While wild type *soxR* was positively associated with reduced susceptibility to minocycline and a synergistic activity with the combination, a negative association was found for *soxR* mutation A111T. This observation aligns with a previous study where this mutation was not associated with resistance to tetracycline or other antibiotics (22).

Further, several sequence variations in genes involved in LPS synthesis or modification showed statistically significant associations with enhanced activity of polymyxin B and minocycline or rifampin in combination (25). These genetic variations might have altered minocycline or rifampin permeability as well as polymyxin B targets. For the minocycline combination, mutant *lpxB* and *lpxK* L323S were associated with synergy, while the C27Y mutation in *eptA* was negatively associated with synergy. LpxB has a role in the addition of a saccharide to the lipid A structure and LpxK catalyzes the addition of the phosphate group. The cation-linkages between phosphates of the lipid A molecules are an important feature for membrane stability and the negatively charged phosphate groups are also a target of polymyxin B (12). Interestingly, minocycline has a potent antioxidant activity and can also directly chelate Ca^2+^ which could also contribute to synergy with polymyxins by displacing the cation-linkages (Ca^2+^ and Mg^2+^) between two lipid A molecules and increase permeability (12, 29). Synergy with polymyxin B and rifampin was positively associated with mutations in *arnT*. Both ArnT and EptA mediate additions of positively charged moieties to the phosphate groups, which could alter polymyxin B activity (25).

**FIG 2:**
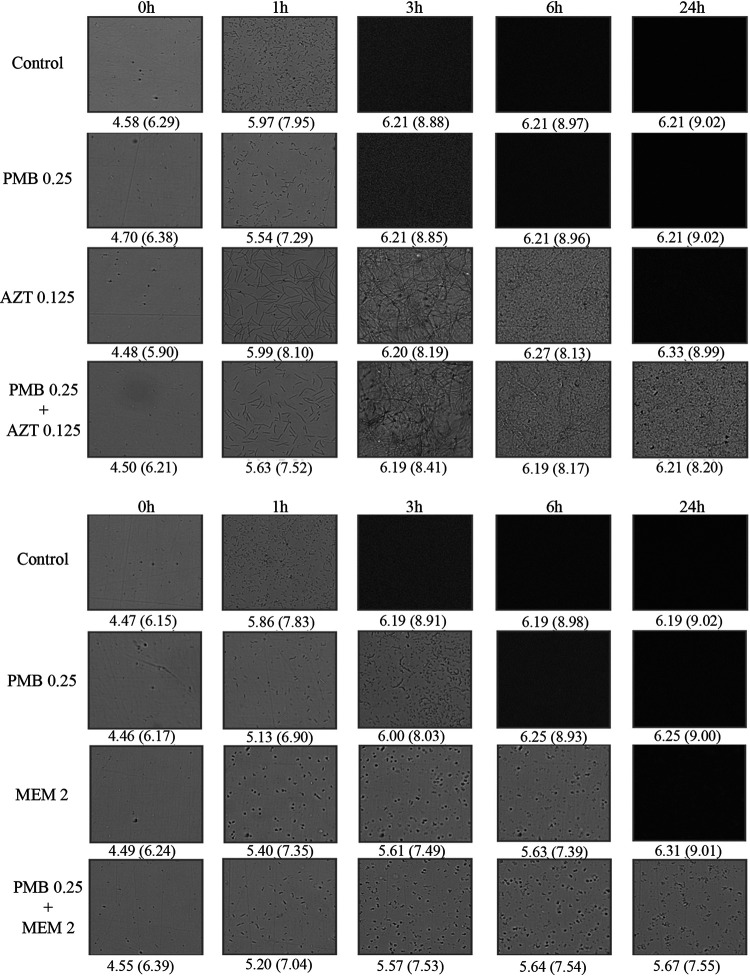
Changes in cell morphology during exposure to polymyxin B (PMB), aztreonam (ATM) and meropenem (MEM) against NDM-producing Escherichia coli ARU786. Antibiotics were added to the indicated concentrations (mg/liter). Images were obtained at 0, 1, 3, 6 and 24 h. The SESA_max_ and BCA (in parentheses) values are presented below each image. Filamentation during exposure to aztreonam alone resulted in high BCA and SESA_max_ values despite low viable counts.

The spot assay added information on CFU/ml reductions and enabled assessment of resistance development during antibiotic exposure. The measurement of bacterial concentrations with this assay is similar to standard time-kill experiments but has lower resolution as individual colonies are not counted (only growth/no growth with a 1:10 dilution between spots) and a higher LOD of 2 log_10_ versus 1 log_10_ CFU/ml. Also, the time-lapse microscopy method differs from time-kill experiments in that there is no shaking during incubation and the total volume is lower (200 μl versus ca 2 ml) (13). The agreement in results between the oCelloScope readout and spot assay was excellent with the exception of aztreonam, for which filamentation complicates readout using the available SESA and BCA algorithms (Fig. 2). Filament formation is associated with β-lactam antibiotics targeting penicillin-binding protein 3 (PBP3), including aztreonam, and was previously observed in time-lapse microscopy experiments with K. pneumoniae and P. aeruginosa (9, 13, 14).

The extensive genetic characterization of resistance mechanisms and mutations, and the assessment of their potential associations with the combination effects is a strength of this study. However, we recognize that more research is needed to validate our findings and determine causality. Combination therapy will remain important in the treatment of multidrug-resistant pathogens to enhance bacterial killing and suppress emergence of resistance, and further efforts to better understand the determinants of synergistic interactions are needed. A range of clinically achievable drug concentrations was used to reduce the risk of overlooking synergistic activity. However, in some cases positive interactions were detected only at the highest drug concentrations, which may be associated with a risk of toxicity in patients. As always, translation of *in vitro* findings to the clinical setting must also be made with caution due to the absence of an immune system and other biological processes as well as differences in growth conditions.

In conclusion, we report positive interactions with polymyxin B combinations against E. coli producing NDM and OXA-48-group carbapenemases, most frequently with minocycline or rifampin. These combinations should be further explored *in vitro* and *in vivo* to determine their therapeutic potential. Resistance genes or mutations involved in efflux, LPS synthesis or modification and lipid A modification were associated with synergistic effect. Deciphering such associations between combination effects and bacterial genetics is a first step toward understanding the mechanisms of synergistic interactions, and may help inform individualized therapy tailored to the infecting pathogen in future patients.

## MATERIALS AND METHODS

### Antibiotics and media.

All antibiotics were purchased from Sigma-Aldrich (St. Louis, MO). Stock solutions of 10,000 mg/liter were prepared by dissolving polymyxin B and meropenem in sterile water and aztreonam, minocycline and rifampin in DMSO. Cation-adjusted Mueller-Hinton (MH-II) (BD Diagnostics, Sparks, MD, USA) broth and agar plates were used for all experiments.

### Strains and antibiotic susceptibility testing.

Twenty carbapenemase-producing E. coli isolates collected from hospitalized patients in Oman during 2015 were used. The susceptibilities to polymyxin B, meropenem, minocycline. and rifampin were tested with broth microdilution according to CLSI recommendations (30). Aztreonam MICs were determined using the Sensititre Antimicrobial Susceptibility Testing System (Trek Diagnostic Systems, Cleveland, OH) according to the manufacturer’s instructions. Susceptibilities were interpreted using CLSI clinical breakpoints M100-ED30:2020 (31).

### Genetic characterization.

DNA was extracted with the MagNA Pure96 System (F. Hoffmann-La Roche, Basel, Switzerland) followed by whole-genome sequencing using HiSeq 2500 (Illumina, San Diego, USA). *De novo* assembly was accomplished using CLC Genomics Workbench (version 20). ResFinder 4.1 was employed to identify acquired resistance genes, (32). Because all strains were susceptible, the search for polymyxin B resistance genes was restricted to *mcr*. To identify variations in genes involved in AcrAB-TolC efflux (*acrA*, *acrB*, *acrR*, *tolC*, *marR*, *marA*, *marB*, *soxS*, *soxR*, *rob)*, porin-specific entry (*ompC*, *ompF*, *ompR*, *envZ*), LPS synthesis (*lpp*, *lpxA-D*, *lpxH*, *lpxK-M*, *lpxP*, *ftsH*, *lapB*, *arnT*, *eptA*, *pagP* and the *waa* locus) and rifampin resistance (*rpoB*) genes were aligned against E. coli MG1655 K-12 (NCBI Reference Sequence: NC_000913.3) and the core oligosaccharide type was determined based on the *waa* locus composition (25).

### Time-lapse microscopy.

Screening was performed using the oCelloScope instrument as previously described (9, 13, 14). Briefly, bacteria in exponential growth phase were added to achieve starting inocula ∼10^6^ CFU/ml and a total volume of 200 µl per well in a flat-bottom 96-well microtiter plate (Greiner Bio-One GmbH, Frickenhausen, Germany). The following clinically achievable drug concentrations were used: polymyxin B, 0.25, 0.5, 1 and 2 mg/liter; aztreonam, 2, 8 and 64 mg/liter; meropenem, 2, 16 and 64 mg/liter; minocycline, 0.5, 4 and 16 mg/liter; and rifampin, 1, 8 and 32 mg/liter. If one of the single antibiotics of a combination prevented bacterial growth at all these concentrations, a lower concentration range was used: polymyxin B, 0.125, 0.25, 0.5 and 1 mg/liter; aztreonam, 0.125, 0.5 and 2 mg/liter; and meropenem, 0.125, 0.5 and 2 mg/liter. Quality control strains (E. coli ATCC 25922 for polymyxin B, aztreonam and meropenem and Staphylococcus aureus ATCC 29213 for minocycline and rifampin) were included in all experiments. The 96-well microtiter plate was incubated at 37°C and images of each well were generated every 15 min for 24 h by the oCelloScope. Focus was set using the bottom search function, illumination level was set to 150, and image distance to 4.9 µm.

The Background Corrected Absorption (BCA) and Segmentation Extracted Surface Area (SESA) algorithms of the UniExplorer software version 6.0.0 (Philips BioCell A/S, Allerød, Denmark) were used to determine bacterial density. The LOD was ∼1 × 10^4^ CFU/ml. A BCA value >8 and a maximum SESA value (SESA_max_) >5.8 were used as cutoff values to indicate a bacterial density of >10^6^ CFU/ml at 24 h (13). The combination was considered to exhibit a positive interaction if BCA and SESA_max_ were below these cutoffs with the combination but not with any of the constituent single antibiotics at the same concentration. Conversely, the combination was considered to show a negative interaction if BCA and SESA_max_ were above the cutoff values with the combination but not with the single antibiotics at the same drug concentrations.

### Spot assay and population analysis.

After completing the 24-h time-lapse microscopy experiments, samples from each well were serially diluted in PBS and 10 μl aliquots were spotted on MH-II agar plates with and without 2 mg/liter polymyxin B (4× MIC) (33). Bacterial growth was recorded after overnight incubation at 37°C. The LOD was 2 log_10_ CFU/ml. No visible bacterial growth was recorded as 1 log_10_ CFU/ml in the analysis of synergistic and bactericidal effects. Synergy was defined as ≥2-log_10_ CFU/ml reduction in bacterial concentrations with the combination at 24 h compared with the most potent single antibiotic (33). A bactericidal effect was defined as ≥ 3-log_10_ reduction in CFU/ml at 24 h compared with the starting inoculum. A ≥1-log_10_ CFU/ml increase in bacterial concentrations with the combination compared to one or both single antibiotics at the same drug concentration was classified as antagonism. Potential antibiotic carryover effects were assessed by regular plating of 100 μl undiluted and 10-fold diluted samples, allowing the sample to sink in before spreading. Two strains producing NDM-1 (ARU770) or OXA-48 (ARU783) were randomly selected for MIC determination of all spots growing on 4× MIC plates after 24 h.

### Statistical analyses.

Potential associations between synergistic effects with an antibiotic combination and the presence of resistance genes and mutations in the tested strains were assessed by Fisher's exact test using R (version 3.6.3). Resistance genes showing statistically significant associations, defined as *P* < 0.05, were further explored to identify correlations between combination interactions and specific mutations in these genes.

### Data availability.

Whole-genome sequencing raw data (reads) were deposited in the Sequence Read Archive (SRA) as project PRJNA544438 (accession numbers SRR9113453, SRR9113455-SRR9113460, SRR9113462, SRR9113468, SRR9113469, SRR9113478-SRR9113487).
