# Supplementary material for: Interactions of Polymyxin B in Combination with Aztreonam, Minocycline, Meropenem, and Rifampin against Escherichia coli Producing NDM and OXA-48-Group Carbapenemases
Source: Antimicrob Agents Chemother. 2021 Nov 17;65(12):e01065-21. doi: 10.1128/AAC.01065-21 (PMC8597741; doi:10.1128/AAC.01065-21)
Supplement: Supplemental file 1 — Supplemental Tables S1 to S4. Download aac.01065-21-s0001.pdf, PDF file, 1.0 MB [file aac.01065-21-s0001.pdf]

Table S1. Statistical analyses[a] of associations between resistance genes and synergy with polymyxin B in combination with minocycline or rifampicin.

	polymyxin B and minocycline synergy					polymyxin B and rifampicin synergy				
	no (n=9)	yes (n=11)	P-value	odds ratio	95 % CI	no (n=11)	yes (n=9)	P-value	odds ratio	95 % CI
tetA+	7	1	0.0045	0.038	0 - 0.51					
tetA-	2	10								
tetB+	1	7	0.0281	**12.052**	1 - 703.94					
tetB-	8	4								
tetD+	0	2	0.4789	Inf	0.155 - Inf					
tetD-	9	9								
*ompC**	9	10	1.0000	0.000	0 - 47.63	11	8	0.450	0.000	0 - 31.91
WT	0	1				0	1			
*ompF**	2	4	0.6424	1.932	0.2 - 28.14	2	4	0.336	3.364	0.34 - 50.19
WT	7	7				9	5			
*acrA**	3	2	0.6169	0.463	0.03 - 5.39	2	3	0.617	2.158	0.19 - 33.41
WT	6	9				9	6			
*acrB**	3	3	1.0000	0.761	0.07 - 7.87	3	3	1.000	1.314	0.13 - 13.74
WT	6	8				8	6			
*acrR**	6	5	0.4059	0.436	0.04 - 3.49	8	3	0.175	0.206	0.02 - 1.74
WT	3	6				3	6			
*tolC**	1	1	1.0000	0.809	0.01 - 70.52	0	2	0.190	Inf	0.24 - Inf
WT	8	10				11	7			
*marR**	4	10	0.0499	**10.805**	0.84 - 645.38	7	7	0.642	1.932	0.2 - 28.14
WT	5	1				4	2			
*marA**	0	2	0.4789	Inf	0.15 - Inf	1	1	1.000	1.236	0.01 - 107.7
WT	9	9				10	8			
*marB**	4	11	0.0081	Inf	1.56 - Inf	7	8	0.319	4.251	0.32 - 252.28
WT	5	0				4	1			
*soxS**	0	1	1.0000	Inf	0.02 - Inf	1	0	1.000	0.000	0 - 47.63
WT	9	10				10	9			
*soxR**	8	3	0.0098	0.057	0 - 0.7	6	5	1.000	1.040	0.13 - 8.61
WT	1	8				5	4			
*rob**	0	1	1.0000	Inf	0.02 - Inf	1	0	0.450	Inf	0.03 - Inf
WT	9	10				10	9			
*ompR**	0	0	-	-	-	0	0	-	-	-
WT	9	11				11	9			
*envZ**	3	2	0.6169	0.463	0.03 - 5.39	2	3	0.617	2.158	0.19 - 33.41
WT	6	9				9	6			

[a] Fisher's exact test; P-values < 0.05 are highlighted in grey and odds ratios ≥ 1 in bold.
Abbreviations: *, amino acid variation; WT, wildtype

Table S2. Statistical analyses[a] of associations between gene mutations and synergy with polymyxin B in combination with minocycline.

	polymyxin B and minocycline synergy				
Gene/mutation	no (n=9)	yes (n=11)	P-value	odds ratio	95 % CI
marR					
S3N	0	1	1.0000	Inf	0.02 - Inf
-	9	10			
K62R	2	1	0.5658	0.369	0.01 - 8.41
-	7	10			
A70E	0	1	1.0000	Inf	0.02 - Inf
-	9	10			
G103S	3	9	0.0648	7.876	0.83 - 123.79
-	6	2			
Y137H	4	9	0.1597	5.108	0.54 - 76.37
-	5	2			
Del97_107	1	0	0.4500	0.000	0 - 31.91
-	8	11			
X	0	1	1.0000	Inf	0.02 - Inf
-	9	10			
marB					
S5L	3	3	1.0000	0.761	0.07 - 7.87
-	6	8			
A10T	0	1	1.0000	Inf	0.02 - Inf
-	9	10			
L12F	2	1	0.5658	0.369	0.01 - 8.41
-	7	10			
A17T	3	1	0.2848	0.217	0 - 3.43
-	6	10			
V20I	3	1	0.2848	0.217	0 - 3.43
-	6	10			
T24P	0	1	1.0000	Inf	0.02 - Inf
-	9	10			
A33G	0	2	0.4789	Inf	0.15 - Inf
-	9	9			
V38A	0	1	1.0000	Inf	0.02 - Inf
-	9	10			
H44Q	4	10	0.04985	**10.8051**	0.84 - 645.38
-	5	1			
X	0	1	1.0000	Inf	0.02 - Inf
-	9	10			
soxR					
T38S	3	1	0.2848	0.217	0 - 3.43
-	6	10			
G74R	3	2	0.6169	0.463	0.03 - 5.39
-	6	9			
A111T	5	1	0.0499	0.093	0 - 1.19
-	4	10			

[a] Fisher's exact test; P-values < 0.05 are highlighted in grey and odds ratios ≥ 1 in bold.
Abbreviations: - , absence of the mutation; Del, deletion; x, gene not found.

Table S3. Statistical analyses[a] of associations between mutations in genes encoding enzymes involved in lipopolysaccharide synthesis or core oligosaccharide type and synergy with polymyxin B and minocycline.

gene/sequence variation	no synergy (n=9)	synergy (n=11)	P-value	odds ratio	95 % CI
lppΔ	1	0	0.4500	Inf	0.03 - Inf
WT	8	11			
lpp_R77H	1	0	0.4500	Inf	0.03 - Inf
-	8	11			
lpxAΔ	0	0	-	-	-
WT	9	11			
lpxCΔ	0	0	-	-	-
WT	9	11			
lpxDΔ	3	2	0.6169	2.16	0.19 - 33.41
WT	6	9			
lpxD_K147R	2	1	0.5658	0.37	0.01 - 8.41
-	7	10			
lpxD_R206C	0	1	1.0000	Inf	0.02 - Inf
-	9	10			
lpxD_I224V	3	2	0.6169	0.46	0.03 - 5.39
-	6	9			
lpxHΔ	4	9	0.1597	0.20	0.01 - 1.84
WT	5	2			
lpxH_V13A	3	2	0.6169	0.46	0.03 - 5.39
-	6	9			
lpxH_R57H	0	1	1.0000	Inf	0.02 - Inf
-	9	10			
lpxH_K58Q	4	9	0.1597	5.11	0.54 - 76.37
-	5	2			
lpxH_R109Q	1	0	0.4500	Inf	0.03 - Inf
-	8	11			
lpxH_T139M	1	3	0.5913	2.85	0.18 - 176.62
-	8	8			
lpxH_W190G	1	2	1.0000	1.73	0.08 - 117.7
-	8	9			
lpxH_P210T	0	1	1.0000	Inf	0.02 - Inf
-	9	10			
lpxBΔ	4	10	0.0499	0.09	0 - 1.19
WT	5	1			
lpxB_E3K	1	4	0.3189	4.25	0.32 - 252.28
-	8	7			
lpxB_H32R	4	7	0.6534	2.10	0.26 - 18.81
-	5	4			

gene/sequence variation	no synergy (n=9)	synergy (n=11)	P-value	odds ratio	95 % CI
lpxB_K84T	1	0	0.4500	Inf	0.03 - Inf
-	8	11			
lpxB_V93I	0	1	1.0000	Inf	0.02 - Inf
-	9	10			
lpxB_A180G	3	3	1.0000	0.76	0.07 - 7.87
-	6	8			
lpxB_H189Y	1	3	0.5913	2.85	0.18 - 176.62
-	8	8			
lpxB_E247A	0	1	1.0000	Inf	0.02 - Inf
-	9	10			
lpxB_R220C	0	1	1.0000	Inf	0.02 - Inf
-	9	10			
lpxB_L256M	3	1	0.2848	0.22	0 - 3.43
-	6	10			
lpxB_M260L	1	0	0.4500	0.00	0 - 31.91
-	8	11			
lpxKΔ	4	10	0.0499	0.09	0 - 1.19
WT	5	1			
lpxK_Q74K	0	1	1.0000	Inf	0.02 - Inf
-	9	10			
lpxK_V66I	1	1	1.0000	0.81	0.01 - 70.52
-	8	10			
lpxK_Y93N	0	1	1.0000	Inf	0.02 - Inf
-	9	10			
lpxK_D118G	2	2	1.0000	0.79	0.05 - 13.47
-	7	9			
lpxK_D225E	1	0	0.4500	0.00	0 - 31.91
-	8	11			
lpxK_A278T	0	3	0.2184	Inf	0.35 - Inf
-	9	8			
lpxK_R293L	1	1	1.0000	0.81	0.01 - 70.52
-	8	10			
lpxK_A294P	1	1	1.0000	0.81	0.01 - 70.52
-	8	10			
lpxK_E298G	1	1	1.0000	0.81	0.01 - 70.52
-	8	10			
lpxK_T319A	3	2	0.6169	0.46	0.03 - 5.39
-	6	9			

gene/sequence variation	no synergy (n=9)	synergy (n=11)	P-value	odds ratio	95 % CI
lpxK_L323S	4	10	0.0499	**10.81**	0.84 - 645.38
-	5	1			
lpxK_N328H	0	1	1.0000	Inf	0.02 - Inf
-	9	10			
lpxLΔ	0	2	0.4789	0.00	0 - 6.47
WT	9	9			
lpxL_M254I	0	1	1.0000	Inf	0.02 - Inf
-	9	10			
lpxL_M185T	0	1	1.0000	Inf	0.02 - Inf
-	9	10			
lpxMΔ	0	2	0.4789	0.00	0 - 6.47
WT	9	9			
lpxM_A98V	0	1	1.0000	Inf	0.02 - Inf
-	9	10			
lpxM_broken	0	1	1.0000	Inf	0.02 - Inf
-	9	10			
R1	3	5	0.6699	1.62	0.2 - 15.65
-	6	6			
R2	3	1	0.2848	0.22	0 - 3.43
-	6	10			
R3	0	3	0.2184	Inf	0.35 - Inf
-	9	8			
R4	3	2	0.6169	0.46	0.03 - 5.39
-	6	9			
ftsH_Δ	0	0	-	-	-
WT	9	11			
lapBΔ	2	2	1.0000	1.27	0.07 - 21.79
WT	7	9			
lapB_scaffold	0	1	1.0000	Inf	0.02 - Inf
-	9	10			
lapB_T201A	2	1	0.5658	0.37	0.01 - 8.41
-	7	10			
lapB_D323N	1	0	0.4500	0.00	0 - 31.91
-	8	11			
lapB_E331K	1	0	0.4500	0.00	0 - 31.91
-	8	11			
lapB_T366A	1	1	1.0000	0.81	0.01 - 70.52
-	8	10			

[a] Fisher's exact test; P-values < 0.05 are highlighted in grey and odds ratios ≥ 1 in bold.
Abbreviations: Δ, amino acid variations; WT, wildtype; -, absence of mutation.

Table S3 (continued).

gene/sequence variation	no synergy (n=9)	synergy (n=11)	p-value	odds ratio	95 % CI	gene/sequence variation	no synergy (n=9)	synergy (n=11)	p-value	odds ratio	95 % CI
arnTΔ	3	8	0.1748	0.21	0.02 - 1.74	*eptA_E547K*	0	2	0.4789	Inf	0.15 - Inf
WT	6	3				-	9	9			
arnT T33M	0	1	1.0000	Inf	0.02 - Inf	*pagPΔ*	3	3	1.0000	1.31	0.13 - 13.74
-	9	10				*WT*	6	8			
arnT_D108N	0	1	1.0000	Inf	0.02 - Inf	*pagP_N2I*	1	0	0.4500	0.00	0 - 31.91
-	9	10				-	8	11			
arnT_T116A	3	6	0.4059	2.29	0.29 - 22.33	*pagP_K5Q*	1	1	1.0000	0.81	0.01 - 70.52
-	6	5				-	8	10			
arnT_T197S	2	5	0.3742	2.76	0.3 - 39.44	*pagP_S11F*	0	1	1.0000	Inf	0.02 - Inf
-	7	6				-	9	10			
arnT_T197M	0	1	1.0000	Inf	0.02 - Inf	*pagP_R35G*	1	0	0.4500	0.00	0 - 31.91
-	9	10				-	8	11			
arnT_N232D	3	8	0.1748	4.85	0.58 - 54.88	*pagP_A39T*	1	0	0.4500	0.00	0 - 31.91
-	6	3				-	8	11			
arnT_L248M	2	5	0.3742	2.76	0.3 - 39.44	*pagP_L82Q*	2	3	1.0000	1.29	0.11 - 19.86
-	7	6				-	7	8			
arnT_V261L	3	7	0.3698	3.27	0.41 - 33.27	*pagP_T117I*	0	1	1.0000	Inf	0.02 - Inf
-	6	4				-	9	10			
arnT_T281A	3	7	0.3698	3.27	0.41 - 33.27	*lpxPΔ*	4	4	1.0000	1.38	0.16 - 11.91
-	6	4				*WT*	5	7			
arnT_S322P	3	6	0.4059	2.29	0.29 - 22.33	*lpxP_scaffold*	0	1	1.0000	Inf	0.02 - Inf
-	6	5				-	9	10			
arnT_P408S	3	5	0.6699	1.62	0.2 - 15.65	*lpxP_Q4K*	2	1	0.5658	0.37	0.01 - 8.41
-	6	6				-	7	10			
arnT_D522N	2	5	0.3742	2.76	0.3 - 39.44	*lpxP_A46T*	0	1	1.0000	Inf	0.02 - Inf
-	7	6				-	9	10			
arnT_A535V	0	1	1.0000	Inf	0.02 - Inf	*lpxP_M47I*	1	0	0.4500	0.00	0 - 31.91
-	9	10				-	8	11			
eptAΔ	9	11	-	-	-	*lpxP_E73K*	0	1	1.0000	Inf	0.02 - Inf
WT	0	0				-	9	10			
eptA_C27Y	5	1	0.0499	0.09	0 - 1.19	*lpxP_G187S*	0	1	1.0000	Inf	0.02 - Inf
-	4	10				-	9	10			
eptA_A147T	1	1	1.0000	0.81	0.01 - 70.52	*lpxP_G125F*	1	0	0.4500	0.00	0 - 31.91
-	8	10				-	8	11			
eptA_K233T	1	1	1.0000	0.81	0.01 - 70.52	*lpxP_G192S*	0	1	1.0000	Inf	0.02 - Inf
-	8	10				-	9	10			
eptA_D348G	4	7	0.6534	2.10	0.26 - 18.81	*lpxP_F253Y*	3	3	1.0000	0.76	0.07 - 7.87
-	5	4				-	6	8			
eptA_T413S	1	6	0.0703	8.53	0.71 - 493.68	*lpxP_T263A*	3	2	0.6169	0.46	0.03 - 5.39
-	8	5				-	6	9			

[a] Fisher's exact test; P-values < 0.05 are highlighted in grey and odds ratios ≥ 1 in bold.

Abbreviations: Δ, amino acid variations; WT, wildtype; -, absence of mutation.

Table S4. Statistical analyses[a] of associations between mutations in genes encoding enzymes involved in lipopolysaccharide synthesis or core oligosaccharide type and synergy with polymyxin B and rifampicin.

gene/sequence variation	no synergy (n=11)	synergy (n=9)	p-value	odds ratio	95 % CI	gene/sequence variation	no synergy (n=11)	synergy (n=9)	p-value	odds ratio	95 % CI	gene/sequence variation	no synergy (n=11)	synergy (n=9)	p-value	odds ratio	95 % CI
lppΔ	1	0	1.000	Inf	0.02 - Inf	*lpxB_K84T*	0	1	0.450	Inf	0.03 - Inf	*lpxK_L323S*	7	7	0.642	1.932	0.2 - 28.14
WT	10	9				-	11	8				-	4	2			
lpp_R77H	1	0	1.000	0.000	0 - 47.63	*lpxB_V93I*	0	1	0.450	Inf	0.03 - Inf	*lpxK_N328H*	1	0	1.000	0.000	0 - 47.63
-	10	9				-	11	8				-	10	9			
lpxAΔ	0	0	-	-	-	*lpxB_A180G*	3	3	1.000	1.314	0.13 - 13.74	*lpxLΔ*	2	0	0.479	Inf	0.15 - Inf
WT	11	9				-	8	6				*WT*	9	9			
lpxCΔ	0	0	-	-	-	*lpxB_H189Y*	2	2	1.000	1.270	0.07 - 21.79	*lpxL_M254I*	1	0	1.000	0.000	0 - 47.63
WT	11	9				-	9	7				-	10	9			
lpxDΔ	2	3	0.617	0.463	0.03 - 5.39	*lpxB_E247A*	1	0	1.000	0.000	0 - 47.63	*lpxL_M185T*	1	0	1.000	0.000	0 - 47.63
WT	9	6				-	10	9				-	10	9			
lpxD_K147R	0	3	0.074	Inf	0.56 - Inf	*lpxB_R220C*	1	0	1.000	0.000	0 - 47.63	*lpxMΔ*	1	1	1.000	0.809	0.01 - 70.52
-	11	6				-	10	9				*WT*	10	8			
lpxD_R206C	1	0	1.000	0.000	0 - 47.63	*lpxB_L256M*	1	3	0.285	4.605	0.29 - 287.54	*lpxM_A98V*	1	0	1.000	0.000	0 - 47.63
-	10	9				-	10	6				-	10	9			
lpxD_I224V	2	3	0.617	2.158	0.19 - 33.41	*lpxB_M260L*	1	0	1.000	0.000	0 - 47.63	*lpxM_broken*	0	1	0.450	Inf	0.03 - Inf
-	9	6				-	10	9				-	11	8			
lpxHΔ	6	7	0.374	0.362	0.03 - 3.31	*lpxKΔ*	7	7	0.642	0.518	0.04 - 5.12	*R1*	6	2	0.197	0.257	0.02 - 2.28
WT	5	2				*WT*	4	2				-	5	7			
lpxH_V13A	2	3	0.617	2.158	0.19 - 33.41	*lpxK_Q74K*	1	0	1.000	0.000	0 - 47.63	*R2*	3	1	0.591	0.351	0.01 - 5.52
-	9	6				-	10	9				-	8	8			
lpxH_R57H	1	0	1.000	0.000	0 - 47.63	*lpxK_V66I*	0	2	0.190	Inf	0.24 - Inf	*R3*	1	2	0.566	2.710	0.12 - 184.85
-	10	9				-	11	7				-	10	7			
lpxH_K58Q	6	7	0.374	2.763	0.3 - 39.44	*lpxK_Y93N*	0	1	0.450	Inf	0.03 - Inf	*R4*	1	4	0.127	7.159	0.53 - 430.99
-	5	2				-	11	8				-	10	5			
lpxH_R109Q	0	1	0.450	Inf	0.03 - Inf	*lpxK_D118G*	1	3	0.285	4.605	0.29 - 287.54	*ftsH_Δ*	0	0	-	-	-
-	11	8				-	10	6				*WT*	11	9			
lpxH_T139M	2	2	1.000	1.270	0.07 - 21.79	*lpxK_D225E*	0	1	0.450	Inf	0.03 - Inf	*lapBΔ*	1	3	0.285	0.217	0 - 3.43
-	9	7				-	11	8				*WT*	10	6			
lpxH_W190G	2	1	1.000	0.578	0.01 - 13.17	*lpxK_A278T*	0	3	0.074	Inf	0.56 - Inf	*lapB_scaffold*	1	0	1.000	0.000	0 - 47.63
-	9	8				-	11	6				-	10	9			
lpxH_P210T	1	0	1.000	0.000	0 - 47.63	*lpxK_R293L*	0	2	0.190	Inf	0.24 - Inf	*lapB_T201A*	0	3	0.074	Inf	0.56 - Inf
-	10	9				-	11	7				-	11	6			
lpxBΔ	7	7	0.642	0.518	0.04 - 5.12	*lpxK_A294P*	0	2	0.190	Inf	0.24 - Inf	*lapB_D323N*	0	1	0.450	Inf	0.03 - Inf
WT	4	2				-	11	7				-	11	8			
lpxB_E3K	2	3	0.617	2.158	0.19 - 33.41	*lpxK_E298G*	0	2	0.190	Inf	0.24 - Inf	*lapB_E331K*	0	1	0.450	Inf	0.03 - Inf
-	9	6				-	11	7				-	11	8			
lpxB_H32R	6	5	1.000	1.040	0.13 - 8.61	*lpxK_T319A*	2	3	0.617	2.158	0.19 - 33.41	*lapB_T366A*	0	2	0.190	Inf	0.24 - Inf
-	5	4				-	9	6				-	11	7			

[a] Fisher's exact test; P-values < 0.05 are highlighted in grey and odds ratios ≥ 1 in bold.

Abbreviations: Δ, amino acid variations; WT, wildtype; -, absence of mutation.

Table S4 (continued).

gene/sequence variation	no synergy (n=11)	synergy (n=9)	p-value	odds ratio	95 % CI	gene/sequence variation	no synergy (n=11)	synergy (n=9)	p-value	odds ratio	95 % CI
arnTΔ	4	7	0.092	0.181	0.01 - 0.61	*eptA_E547K*	2	0	0.479	0.000	0 - 6.47
WT	7	2				-	9	9			
arnT_T33M	1	0	1.000	0.000	0 - 47.63	pagPΔ	3	3	1.000	0.761	0.07 - 7.87
-	10	9				*WT*	8	6			
arnT_D108N	1	0	1.000	0.000	0 - 47.63	*pagP_N2I*	1	0	1.000	0.000	0 - 47.63
-	10	9				-	10	9			
arnT_T116A	2	7	0.022	**13.054**	1.28 - 236.66	*pagP_K5Q*	0	2	0.190	Inf	0.24 - Inf
-	9	2				-	11	7			
arnT_T197S	1	6	0.017	**16.482**	1.29 - 1000.51	*pagP_S11F*	1	0	1.000	0.000	0 - 47.63
-	10	3				-	10	9			
arnT_T197M	1	0	1.000	0.000	0 - 47.63	*pagP_R35G*	1	0	1.000	0.000	0 - 47.63
-	10	9				-	10	9			
arnT_N232D	4	7	0.092	5.538	0.62 - 80.98	*pagP_A39T*	1	0	1.000	0.000	0 - 47.63
-	7	2				-	10	9			
arnT_L248M	1	6	0.017	**16.482**	1.29 - 1000.51	*pagP_L82Q*	2	3	0.617	2.158	0.19 - 33.41
-	10	3				-	9	6			
arnT_V261L	3	7	0.070	8.153	0.88 - 127.06	*pagP_T117I*	1	0	1.000	0.000	0 - 47.63
-	8	2				-	10	9			
arnT_T281A	3	7	0.070	8.153	0.88 - 127.06	*lpxPΔ*	3	5	0.362	0.320	0.03 - 2.67
-	8	2				*WT*	8	4			
arnT_S322P	2	7	0.022	**13.054**	1.28 - 236.66	*lpxP_scaffold*	0	1	0.450	Inf	0.03 - Inf
-	9	2				-	11	8			
arnT_P408S	1	7	0.005	**26.599**	1.98 - 1701.87	*lpxP_Q4K*	1	2	0.566	2.710	0.12 - 184.85
-	10	2				-	10	7			
arnT_D522N	1	6	0.017	**16.482**	1.29 - 1000.51	*lpxP_A46T*	1	0	1.000	0.000	0 - 47.63
-	10	3				-	10	9			
arnT_A535V	1	0	1.000	0.000	0 - 47.63	*lpxP_M47I*	0	1	0.450	Inf	0.03 - Inf
-	10	9				-	11	8			
eptAΔ	11	9	-	-	-	*lpxP_E73K*	1	0	1.000	0.000	0 - 47.63
WT	0	0				-	10	9			
eptA_C27Y	4	2	0.642	0.518	0.04 - 5.12	*lpxK_G187S*	1	0	1.000	0.000	0 - 47.63
-	7	7				-	10	9			
eptA_A147T	2	0	0.479	0.000	0 - 6.47	*lpxP_G125F*	1	0	1.000	0.000	0 - 47.63
-	9	9				-	10	9			
eptA_K233T	0	2	0.190	Inf	0.24 - Inf	*lpxP_G192S*	0	1	0.450	Inf	0.03 - Inf
-	11	7				-	11	8			
eptA_D348G	4	7	0.092	5.538	0.62 - 80.98	*lpxP_F253Y*	2	4	0.336	3.364	0.34 - 50.19
-	7	2				-	9	5			
eptA_T413S	3	4	0.642	2.052	0.23 - 20.76	*lpxP_T263A*	1	4	0.127	7.159	0.53 - 430.99
-	8	5				-	10	5			

[a] Fisher's exact test; P-values < 0.05 are highlighted in grey and odds ratios ≥ 1 in bold.

Abbreviations: Δ, amino acid variations; WT, wildtype; -, absence of mutation.
